# Supplementary material for: Dynamic and asymmetric colloidal molecules
Source: Nat Commun. 2025 Mar 21;16:2819. doi: 10.1038/s41467-025-58057-1 (PMC11928658; doi:10.1038/s41467-025-58057-1)
Supplement: Supplementary file 1 — Supplementary Information [file 41467_2025_58057_MOESM1_ESM.pdf]

Supplementary Information for "Dynamic and asymmetric colloidal molecules"

Huang Fang[a,1] Qiong Gao[a,1] Yujie Rong[a,1] Yanshuang Chen,[1] Jiping Huang,[1,2]
Hua Tong,[3] Zhihong Nie,[4,5,*] Hajime Tanaka,[6,7,†] Wei Li,[5,8,‡] and Peng Tan[1,9,§]

[1]*Department of Physics and State Key Laboratory of Surface Physics, Fudan University, Shanghai, 200438, P. R. China*
[2]*Key Laboratory of Micro and Nano Photonic Structures (MOE), Fudan University, Shanghai, 200438, P. R. China*
[3]*Department of Physics, University of Science and Technology of China, Hefei, 230026, P. R. China*
[4]*Department of Macromolecular Science, Fudan University, Shanghai, 200438, P. R. China*
[5]*State Key Laboratory of Molecular Engineering of Polymers, Fudan University, Shanghai, 200438, P. R. China*
[6]*Research Center for Advanced Science and Technology, University of Tokyo, 4-6-1 Komaba, Meguro-ku, Tokyo 153-8904, Japan*
[7]*Department of Fundamental Engineering, Institute of Industrial Science, University of Tokyo, 4-6-1 Komaba, Meguro-ku, Tokyo 153-8505, Japan*
[8]*Department of Chemistry and Laboratory of Advanced Materials, Fudan University, Shanghai 200438, P. R. China*
[9]*Institute for Nanoelectronic Devices and Quantum Computing, Fudan University, Shanghai, 200438, P. R. China*

[*] znie@fudan.edu.cn
[†] tanaka@iis.u-tokyo.ac.jp
[‡] weilichem@fudan.edu.cn
[§] tanpeng@fudan.edu.cn
[a] These authors contributed equally.

SUPPLEMENTARY FIGURES

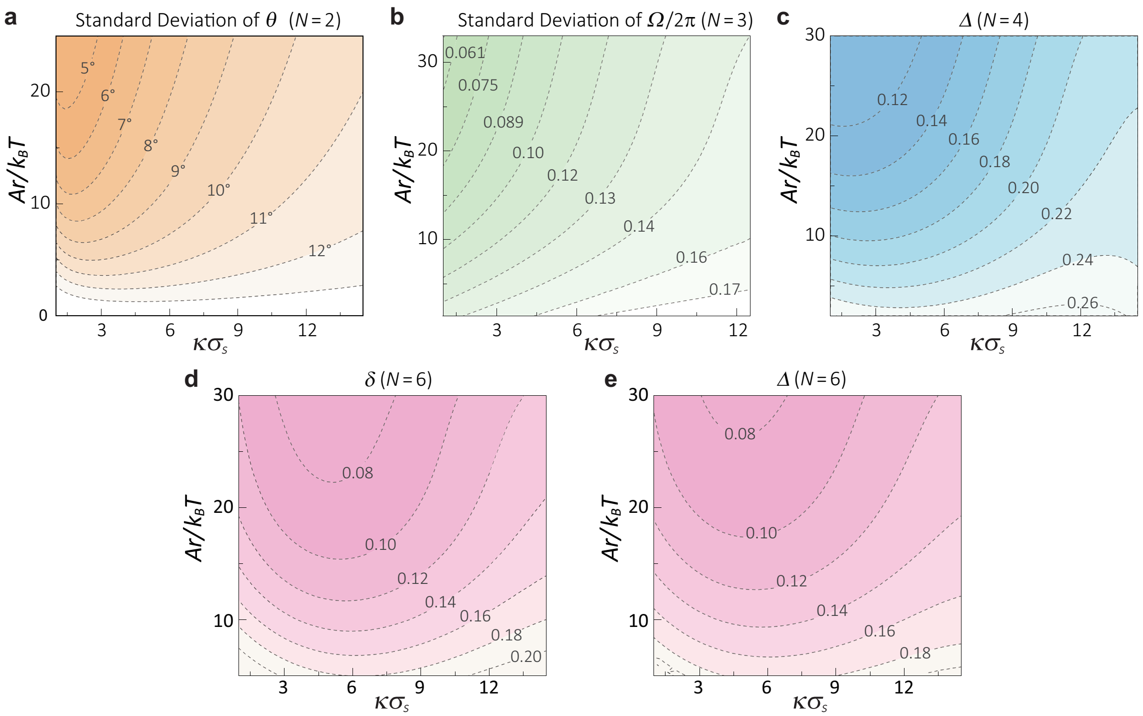

FIG. S1. **Asymmetric structures and configurational fluctuations of N-CMs as a function of $\kappa\sigma_s$ and σ_c. a,** Standard deviations of θ for 2-CMs. **b,** Standard deviations of Ω for 3-CMs.. **c,** The magnitude of configurational fluctuation around the average configuration Δ for 4-CMs. **d** and **e**, δ (in **d**) and Δ(in **e**) for 6-CMs, respectively.

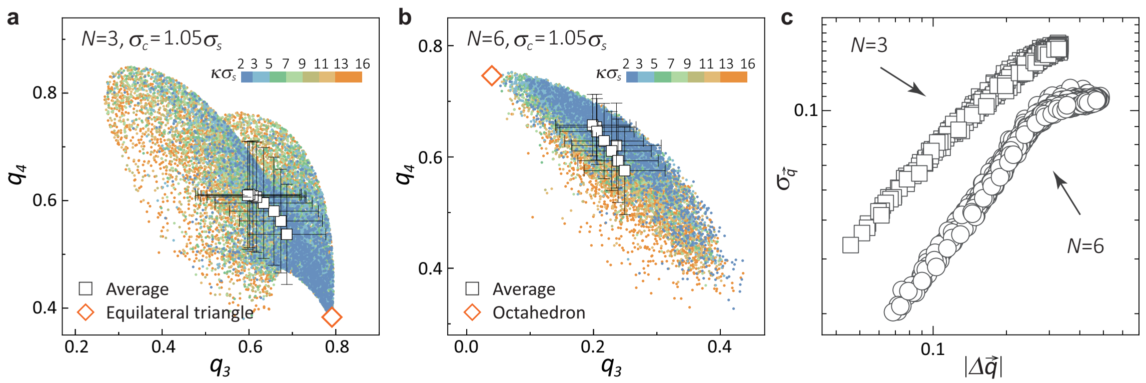

FIG. S2. **Angular orders of 3-CMs and 6-CMs with respect to $\kappa\sigma_s$ and σ_c. a** and **b,** Illustration of the angular order development for 3- and 6-CMs using q_3 and q_4. Square symbols with error bars represent the average and standard deviation (at $\sigma_c = 0.95\ \sigma_s$ and $A_r = 10\ k_BT$). **c,** The $|\Delta\vec{q}|$-$\sigma_{\vec{q}}$ master curve for 3-CMs and 6-CMs for varying A_r (5 to 75 k_BT), σ_c (0.95 to 1.45 σ_s), and $\kappa\sigma_s$ (2 to 30).

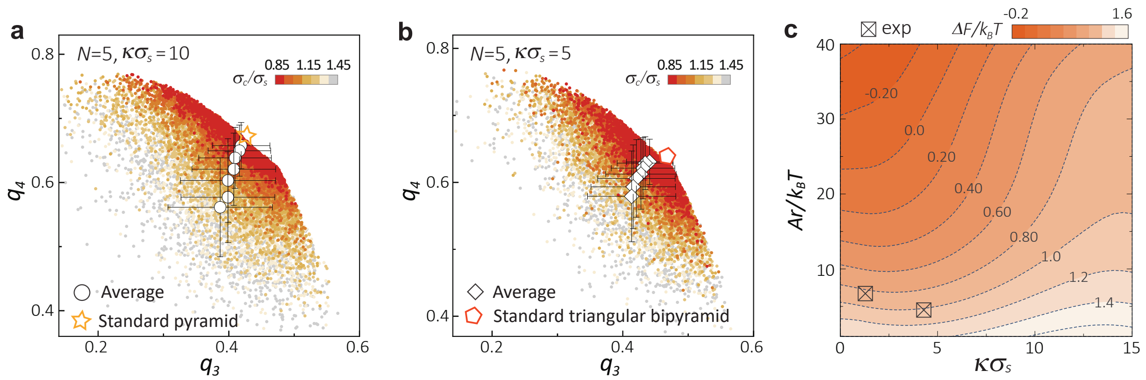

FIG. S3. **Illustration of the bifurcation pathways of 5-CMs with respect to $\kappa\sigma_s$ and σ_c. a** and **b,** Demonstration of the angular order development for 5-CMs through q_3 and q_4. **a** corresponds to $A_r = 90\ k_BT$ and $\kappa\sigma_s = 10$, while **b** corresponds to $A_r = 30\ k_BT$ and $\kappa\sigma_s = 5$. **c,** The free energy difference, ΔF, defined as $(F_T - F_P)/k_BT$, between the triangular bipyramid and pyramid configurations for varying σ_c and $\kappa\sigma_s$.

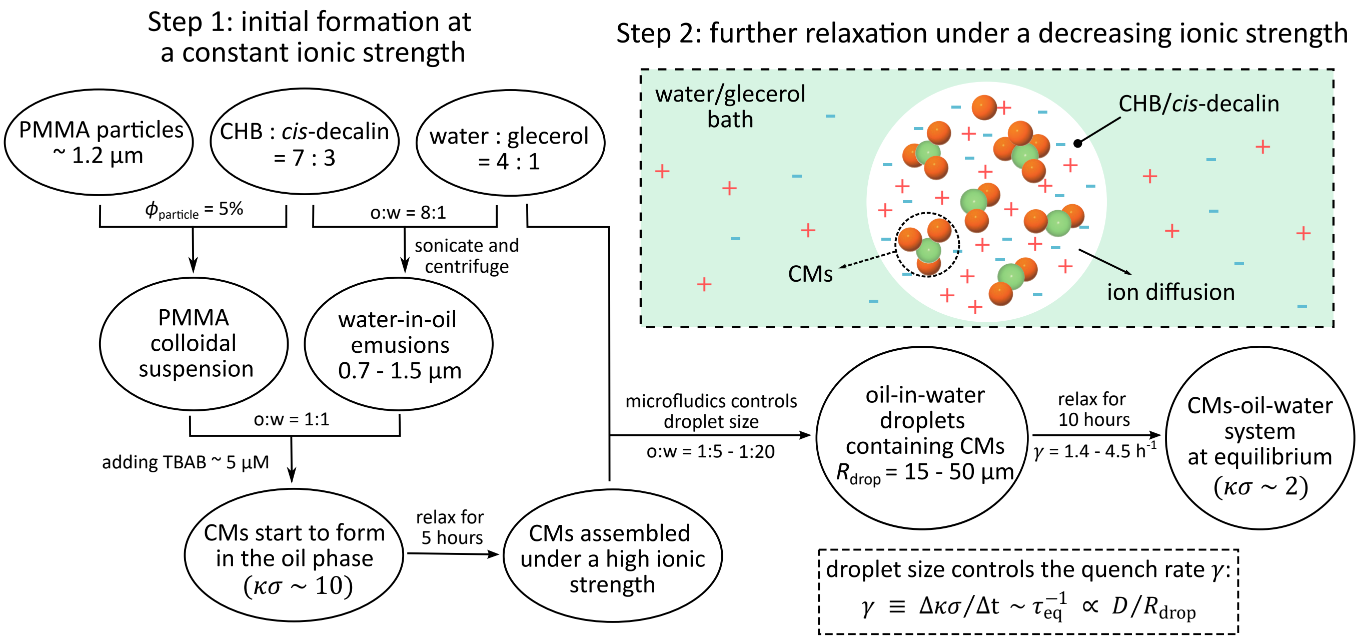

FIG. S4. Flow chart of an experimental plan that implements the quenching protocol. First, CMs are prepared in the oil phase at a high constant ionic strength, as detailed in the Methods section. Next, the oil phase is combined with an aqueous bath to form oil-in-water droplets containing CMs. Due to the ionic strength difference between the oil and water phases, ions diffuse from the oil into water, causing $\kappa\sigma$ to decrease from approximately 10 to 2 over several hours [1]. The quench rate γ is inversely proportional to the equilibration time $\tau_{\rm eq}$: $\tau_{\rm eq} \sim \Delta c V_{\rm drop}/J S_{\rm drop} \propto R_{\rm drop}/D$, where Δc represents the salt concentration difference between the initial and final states of the oil droplet, J is the diffusive flux (proportional to the ion diffusion constant D), and $R_{\rm drop}$ is the droplet radius. By using a microfluidic device, droplet size can be adjusted by tuning the oil-to-water ratio, and thereby controlling the quench rate.

SUPPLEMENTARY REFERENCES

[1] Chen, Y. *et al.* Morphology selection kinetics of crystallization in a sphere. *Nature Physics* **17**, 121–127 (2021).
