# Supplementary material for: Dynamic and asymmetric colloidal molecules
Source: Nat Commun. 2025 Mar 21;16:2819. doi: 10.1038/s41467-025-58057-1 (PMC11928658; doi:10.1038/s41467-025-58057-1)
Supplement: Supplementary file 2 — Description of Additional Supplementary Files [file 41467_2025_58057_MOESM2_ESM.docx]

Description of Additional Supplementary Files

**File Name:** Supplementary Movie 1

**Description:** A N=2 colloidal molecule under confocal microscopy and the reconstructed 3D image of its dynamic trajectory.

**File Name:** Supplementary Movie 2

**Description:** A N=3 colloidal molecule under confocal microscopy and the reconstructed 3D image of its dynamic trajectory.

**File Name:** Supplementary Movie 3

**Description:** A N=4 colloidal molecule under confocal microscopy and the reconstructed 3D image of its dynamic trajectory.

**File Name:** Supplementary Movie 4

**Description:** A N=5 colloidal molecule under confocal microscopy and the reconstructed 3D image of its dynamic trajectory.

**File Name:** Supplementary Movie 5

**Description:** A N=6 colloidal molecule under confocal microscopy and the reconstructed 3D image of its dynamic trajectory.
